# Draft Genome Sequence of Uncultivated *Firmicutes* (*Peptococcaceae* SCADC) Single Cells Sorted from Methanogenic Alkane-Degrading Cultures

**DOI:** 10.1128/genomeA.00909-14

**Published:** 2014-09-11

**Authors:** BoonFei Tan, Rhianna Charchuk, Carmen Li, Camilla Nesbø, Nidal Abu Laban, Julia Foght

**Affiliations:** a Department of Biological Sciences, University of Alberta, Edmonton, Alberta, Canada; b Centre for Ecological and Evolutionary Synthesis, University of Oslo, Oslo, Norway

## Abstract

The draft genome of an uncultivated bacterium affiliated with the *Peptococcaceae* was reconstructed by co-assembling Illumina MiSeq sequences from three single cells sorted by microfluidics from two methanogenic alkane-degrading cultures. *Peptococcaceae* SCADC (short-chain alkane-degrading culture) may be genetically capable of anaerobic alkane activation by fumarate addition in the absence of sulfate.

## GENOME ANNOUNCEMENT

We previously described a methanogenic SCADC (short-chain alkane-degrading culture) enriched from an oil sands tailings pond (1). During incubation at ~25° C and several passages under strictly methanogenic conditions for >2 years, SCADC produced methane with stoichiometric depletion of *n*-hexane, -heptane, -octane, -decane, 2-methylpentane, and methylcyclopentane (1). Subsamples of SCADC and a methanogenic *iso*-alkane-degrading culture (ISODC; N. Abu Laban, A. Dao, K. Semple, J. Foght, submitted for publication), incubated under parallel conditions, were subjected to microfluidic single-cell sorting (https://www.bigelow.org). Two cells from SCADC and one from ISODC were subjected to multiple displacement amplification, sequenced as single-amplified genomes (SAGs) using Illumina MiSeq (http://tagc.med.ualberta.ca/), and individually assembled *de novo* using SPAdes version 3.1 (2) with a read correction module and k-mer sizes of 21, 33, 45, 55, 77, 99, and 127. *De novo* assembly for each sorted cell produced 200 to 206 contigs of 1,000-bp minimum length. Two-way nucleotide identity analysis (http://enve-omics.ce.gatech.edu/ani) of the three genomes yielded an average of 4,136 fragments (500-bp window read size) having 99.24% mean identity and sharing identical 16S rRNA genes. Based on this similarity, all Illumina reads from the three cells were co-assembled *de novo* using SPAdes (2), as above.

To exclude contaminating sequences, scaffolds were fragmented *in silico* to 500 bp followed by BLASTx searches against the NCBI NR database. Fragments were assigned to taxa using MEGAN version 5.0 (3) with a minimum bit-score support of 100. Contigs with >50% of their fragments having hits to *Firmicutes* sequences were retained; the remainder were defined as having weak taxon support. All contigs were then subjected to tetranucleotide frequency analysis followed by clustering (4) in R (http://www.r-project.org). Contigs with weak taxon support that did not cluster with the *Firmicutes*-affiliated contigs were removed from the final genomic bin. The draft genome was named *Peptococcaceae* SCADC (i.e., identified at the family level) because phylogenetic and BLASTn analysis of the 16S rRNA gene sequence showed ≤90% identity to *Desulfotomaculum* and *Pelotomaculum* (the closest cultivated matches), precluding classification to the genus level.

The *Peptococcaceae* SCADC draft genome is ~2.6 Mbp contained in 259 scaffolds with an *N*_50_ of 23,229 bp and an average 42.9% GC content. Annotation using NCBI Prokaryotic Genome Annotation Pipeline predicted 2,208 coding sequences. Ninety-five single-copy genes were recovered using the gene list compiled by Albertsen et al. (5) versus 108 and 109 genes detected in *Pelotomaculum thermopropionicum* SI (NC_009454.1) and *Desulfotomaculum acetoxidans* DSM 771 (NC_013216.1), suggesting that the draft genome is >87% complete.

Genome annotation and phylogenetic analysis detected a putative gene encoding AssA, 47% similar to the AssA subunit in *Desulfoglaeba alkanexedens* ALDC (ADJ51097). Therefore, *Peptococcaceae* SCADC also may be capable of alkane addition to fumarate during anaerobic hydrocarbon degradation. However, whereas *Desulfotomaculum* spp. harbor *dsrAB* genes and reduce sulfate and sulfite (6), the *Peptococcaceae* SCADC draft genome lacks obvious *dsrAB* and *dsrMKJOP* orthologs. Therefore, like *Pelotomaculum* (7), it likely is incapable of complete sulfate reduction. Genomic and metatranscriptomic analyses of *Peptococcaceae* SCADC in alkane-degrading enrichment cultures (1) is under way to study its potential role in alkane degradation under methanogenic conditions.

### Nucleotide accession numbers.

This whole-genome shotgun project has been deposited at DDBJ/EMBL/GenBank under the accession number JJNX00000000. The draft genome described in this paper is version JJNX02000000.
